# Association Between Barthel Index and Delirium During Hospitalization in Older Patients with Acute Decompensated Heart Failure

**DOI:** 10.3390/jcdd13090429

**Published:** 2026-09-01

**Authors:** Michiaki Nagai, Keigo Dote, Masaya Kato, Noboru Oda, Sophie Chang, Phillip J. Tully, Tarun Dasari

**Affiliations:** 1 Department of Internal Medicine, Hiroshima Asa Medical Association Hospital, 2-1-38, Kabeminami, Aaskita-ku, Hiroshima 7310223, Japan; dote1958@gmail.com; 2 Cardiovascular Section, Department of Medicine, University of Oklahoma Health Science Center, Andrews Academic Tower, Suite 5400, Oklahoma City, OK 73104, USA; sophie-chang@ou.edu; 3 Department of Cardiology, Hiroshima City Asa Hospital, 2-1-1, Kabeminami, Aaskita-ku, Hiroshima City Asa, Hiroshima 7310293, Japan; ms-katou@asa-hosp.city.hiroshima.jp; 4 Department of Cardiovascular Medicine, Graduate School of Biomedical and Health Sciences, Hiroshima University, 1-2-3, Kasumi, Minami-ku, Hiroshima 7348551, Japan; odanoboru@mail.goo.ne.jp; 5 Faculty of Medicine and Health, School of Psychology, Deakin University, 221 Burwood Highway, Burwood, VIC 3125, Australia; p.tully@deakin.edu.au; 6 Centre for Men’s Health and Wellbeing, School of Medicine, Faculty of Medicine and Health, Adelaide University, Adelaide, SA, Level 6 AHMS Bldg, Adelaide, SA 5005, Australia

**Keywords:** activity of daily living, delirium, acute decompensated heart failure, older patients, heart failure with preserved ejection fraction

## Abstract

Background: Although delirium is an acute confusional state, the available literature identifies impaired activities of daily living (ADL) as a predisposing factor. However, the question of whether ADL is associated with delirium in older patients with heart failure (HF) has not been evaluated. This study investigated the relationship between ADL and delirium and further explored whether HF phenotype moderates the relationship in older patients with acute decompensated HF (ADHF). Methods: In older patients with ADHF, stabilized after HF admission, handgrip strength (HGS) and 6 min walk distance (6MWD) tests were performed, and ADL was assessed using the Barthel index. Delirium during hospitalization was defined according to the *Diagnostic and Statistical Manual of Mental Disorders, Fifth Edition*. Logistic regression analyses were used to estimate the association of physical activity and ADL with delirium during hospitalization in older patients with ADHF. Results: A total of 245 older patients (82.9 ± 6.0 years, 49.4% male) with ADHF (heart failure with preserved ejection fraction [HFpEF]; n = 107, 44%) were analyzed. In the group with delirium (n = 65, 26.5%), there was significant difference in Mini–Mental state examination (MMSE) score (19 vs. 25, *p* < 0.001), HGS (13 vs. 16 kg, *p* < 0.05), 6MWD (120 vs. 160 m, *p* < 0.05) and Barthel index (55 vs. 65, *p* < 0.05) compared to the group without delirium. In the HFpEF group, 6MWD (*p* < 0.05) and Barthel index (*p* < 0.001) had significant negative correlations with delirium. In the multiple logistic regression analysis adjusted for the confounders, Barthel index (Odds ratio: 0.390 per 1 standard deviation, *p* < 0.01) was significantly associated with delirium in the HFpEF group. Conclusions: In older ADHF patients, decreased physical activity and ADL were significantly correlated with delirium. Lower Barthel index was associated with delirium, and this association was significantly modified by HFpEF status.

## 1. Introduction

Delirium is an acute confusional state characterized by impaired consciousness and altered cognitive function that is common in hospitalized patients, especially older patients [1]. Several studies have demonstrated that the development of delirium during hospitalization is associated with poorer outcomes in postoperative patients [2,3]. Furthermore, it has been suggested that not only increasing age but also decreased physical activity and inability to complete activities of daily living (ADL) increase the likelihood of delirium [4,5].

Acute decompensated heart failure (HF) (ADHF) is a serious medical condition that requires intensive medical support, and the majority of ADHF patients are older, with age a leading risk factor for developing delirium [6]. In ADHF, one in four patients develop delirium, and the incidence of all-cause death is significantly higher in patients with delirium than those without delirium [6]. HF is known to alter the function of skeletal muscle contractile units, and patients with chronic disease typically experience muscle atrophy and weakness, reduced physical activity levels, and, consequently, an inability to independently perform self-care [7]. The ADL ability of HF patients is closely related to prognosis, and one study showed that patients with ADL impairment had a nearly three-fold higher rate of readmission within 3 months compared to patients without ADL impairment [8]. Another study showed that ADL ability was an independent predictor of all-cause mortality in patients with HF [9]. From the above, it is possible that a decline in physical activity and ADL is involved in the onset of delirium during hospitalization and may reflect dimensions of delirium vulnerability that are distinct from those captured by previously identified associated factors. However, few studies have been conducted to investigate the relationships of declines in physical activity and ADL with delirium in ADHF. In this study, we evaluated the hypothesis that reduced physical activity and ADL are associated with delirium in older patients with ADHF and further explored whether HF phenotype moderates the relationship.

## 2. Materials and Methods

### 2.1. Study Population

The study design in this analysis was conducted at a medium tertiary hospital in Japan. We considered patients eligible for study enrollment if they were hospitalized for treatment of ADHF in our department, defined as the rapid onset or worsening of symptoms and/or signs of HF. The HF symptoms were fatigue, breathlessness, and ankle swelling. The signs were: peripheral edema, elevated jugular venous pressure, and pulmonary crackles [10,11]. Each patient’s diagnosis of ADHF was based on a thorough medical history and assessment of the patient’s symptoms, potential cardiac and noncardiac precipitants, and any prior cardiovascular history; the diagnosis also considered the signs/symptoms of hypoperfusion or congestion revealed by a physical examination and confirmed by appropriate additional investigations, i.e., laboratory assessments (including the measurement of B-type natriuretic peptide [BNP]), echocardiography, chest X-ray, and electrocardiography [12]. Left ventricular (LV) ejection fraction (EF) (LVEF) at the time of diagnosis was determined by the Teichholz method or modified Simpson method. HF with reduced EF (HFrEF), HF with mildly reduced EF (HFmrEF) and HF with preserved EF (HFpEF) were defined as LVEF < 40%, 40–49% and ≥50%, respectively [11].

Regarding the patients’ BNP levels used for the diagnosis of ADHF, we set an exclusion cut-off point at 100 pg/mL [12]. We also excluded patients with septic multiple organ failure, septic shock, or sepsis, and those who were on chronic hemodialysis. The consecutive eligible older patients with ADHF over 70 years of age were enrolled after the study’s purpose was fully explained to the patients and their informed consent was obtained. Blood sampling was performed at admission, and cognitive function, physical activity and ADL assessment were simultaneously performed after admission when symptoms disappeared during hospitalization. M.K. and N.O. performed these assessments blinded to the information regarding delirium. Signed informed consent was obtained from all patients in a stable non-delirious state or legally authorized representatives. For participants who were unable to provide informed consent because of cognitive impairment, informed consent was obtained from a legally authorized representative in accordance with institutional ethical guidelines. Participant confidentiality was protected through de-identification of study records and secure storage of data according to institutional policies. In accordance with the Helsinki declaration, the studies involving human participants were reviewed and approved by the Ethics Committees of a local hospital (04119).

### 2.2. Measurement of Physical Activity and Activities of Daily Living

Once HF condition stabilized, physical activity and ADL were assessed with information regarding delirium blinded to the assessors. Depending on the timing of delirium onset, assessment could occur either before or after the development of delirium. In the handgrip strength (HGS) test, patients were required to squeeze the handle maximally and to sustain this for 1–2 s. All measurements were performed for both hands. Subjects performed three maximum attempts for each grip strength measurement, and the mean value of these trials was recorded [13]. The 6MWD is a submaximal exercise test used in the assessment of functional capacity [14]. The 6MWD was performed according to the American Thoracic Society guidelines in a straight, 30 m long corridor under the guidance of a physiotherapist [15]. For this test, participants were instructed to walk at a self-selected pace with the aim of covering as much distance as possible in 6 min. The patient was told to be calm, and to wear comfortable clothing and shoes. Once the patient understood the instructions, the test was ready to begin. During the test, participants had to walk at a speed appropriate to their condition, and they were allowed to stop and slow down if necessary. The supervisor stays with the patient throughout the procedure and encourages them with standard phrases such as “You’re doing well” and “Good work”. Functional status was assessed using the Japanese version of the Barthel index. Assessments were performed by trained nurses/physical therapists. The Barthel index is a cumulative score calculated by summing each item: feeding, bathing, grooming, dressing, bowel control, bladder control, toileting, chair transfer, ambulation, and stair climbing. The Barthel index scores are multiples of 5 with a range of 0 (completely dependent) to 100 (independent in basic ADL). Higher scores represent a higher degree of independence [16]. Ceiling and floor effects were assessed by calculating the proportions of participants who achieved the highest and lowest possible Barthel index scores. Ceiling or floor effects were considered present if more than 15% of participants achieved the maximum or minimum score, respectively.

### 2.3. Assessment and Management of Delirium During Hospitalization and Cognitive Assessment

The presence of delirium was assessed every day by a doctor or a trained delirium specialist including a certified nurse from admission to discharge using the *Diagnostic and Statistical Manual of Mental Disorders, fifth edition (DSM–5)* [17]. All assessors were blinded to information of HGS, 6MWD and Barthel index scores. Consultant liaison psychiatrists were consulted if necessary. Depending on the cases, in collaboration with anesthesiologists, some patients receiving noninvasive positive pressure ventilation received intravenous sedation for respiratory management. If a patient became delirious, delirium was managed sometimes in consultation with a psychiatrist as follows. First, nonpharmacological treatment for delirium was offered to all patients. These included reorientation and environmental interventions such as an appropriate patient care environment with low levels of lighting and minimal noise to avoid sleep interruptions at night. When nonpharmacological treatments were insufficient, additional pharmacological treatments, such as oral antipsychotics, were considered and implemented. In cases of severe agitation, temporary physical restraints were introduced in accordance with clinical practice guidelines [18]. Regardless of study participation, ethical approval and written informed consent by the patient, their family member, or a surrogate was mandatory for restraints, and were performed at the time of admission as routine clinical practice at our institution. Any kind of restraint tended to be discussed by the medical staff in charge, such as cardiologists, psychiatrists, anesthesiologists and nurses, before being introduced to the patient. General cognitive function was assessed using the Mini–Mental state examination (MMSE) [19].

### 2.4. Statistical Analysis

The data are presented as the mean ± the standard deviation, median (interquartile range) or as a percentage, and all analyses were performed with IBM SPSS ver. 28 software (IBM Corp., Armonk, NY, USA). By computing the Kolmogorov–Smirnov test, continuous variables were analyzed to detect substantial deviations from normality. We performed an analysis of variance (ANOVA) for the parametric variables, and the Kruskal–Wallis test was used for non-parametric variables among groups. For the multiple pairwise comparisons of means or proportions between pairs of groups, Tukey’s honestly significant differences test or Mann–Whitney U test was performed. The correlations between variables were determined by univariate regression analysis. To estimate and test the independent effects of physical activity and ADL on delirium, multiple logistic regression analysis was conducted. In addition to age, gender and body mass index (BMI), each baseline model mainly included factors that correlated significantly with delirium as determined by univariate regression model. Receiver operating characteristic (ROC) curves were constructed and the area under the ROC curve (AUC) was calculated in order to study the discriminatory power of physical status and ADL for delirium. The optimal cut-off points for the indication of delirium were derived using the Youden index. The optimal cut-off value was determined based on the Youden’s index (sensitivity + specificity − 1), which maximizes the sum of sensitivity and specificity. Probability (*p*)-values < 0.05 were accepted as significant.

## 3. Results

Values of HGS, 6MWD and Barthel index score were available for 245 enrolled patients. During hospitalization, delirium was observed in 65 patients (26.5%) with ADHF. The majority (n = 61, 93.9%) of delirium cases occurred within 72 h of hospitalization. In the delirium group, 62 patients were classified as hyperactive type and three as hypoactive type, and psychiatrists were consulted in 32.8% of hyperactive type cases and in all cases of hypoactive type. In the groups with delirium and without delirium, the mean age and proportion of females were 83.3 and 82.8 years, and 50.8 and 50.6%, respectively. In addition, there was significant difference in mineralocorticoid receptor antagonists (MRA) use (6.2 vs. 17.2%, *p* < 0.05), prevalence of HFmrEF (32.3 vs. 19.6%, *p* < 0.05), MMSE score (19 vs. 25, *p* < 0.001), HGS (13 vs. 16 kg, *p* < 0.05), 6MWD (120 vs. 160 kg, *p* < 0.05) and Barthel index (55 vs. 65, *p* < 0.05) between the two groups classified by delirium (Table 1). Five participants (2.0%) achieved the maximum Barthel index score, and 11 participants (4.5%) achieved the minimum score. Therefore, neither ceiling nor floor effects were observed. No patients were receiving left ventricular assist device or mechanical circulatory support at the time of assessment of physical activity and ADL. There was almost no missing data. Therefore, imputation was unnecessary, and the data is considered suitable for analysis.

### 3.1. Associations of Physical Activity and ADL with Delirium in Univariate Logistic Regression Analysis

Table 2 summarizes the associations of physical activity and ADL with delirium in the univariate logistic regression analysis. In the total population, MRA use, MMSE score, HGS, 6MWD and Barthel index had significant associations with delirium (Table 2). In the HFrEF group, diabetes mellitus and E/e′ had significant associations with delirium (Table 2). In the HFmrEF group, ejection fraction, MMSE score and HGS had significant associations with delirium (Table 2). In the HFpEF group, MMSE score, 6MWD and Barthel index had significant associations with delirium (Table 2).

### 3.2. Determinants of Delirium

To formally assess effect modification by HF phenotype, interaction terms between Barthel index and HF categories were included in the multivariable logistic regression model. When the HFrEF group is defined as the reference group, a significant interaction was observed between Barthel index and HFpEF status (*p* for interaction = 0.03), whereas no significant interaction was observed for HFmrEF (*p* = 0.40). The variance inflation factor values for all variables in all models were below 1.4, indicating that multicollinearity was not a concern.

In the multiple logistic regression analysis adjusted for the possible confounders including age, gender, BMI, diabetes mellitus, E/e′ and MMSE score, neither HGS, 6MWD nor Barthel index was significantly associated with delirium in HFrEF group. In the HFmrEF group, neither HGS, 6MWD nor Barthel index was significantly associated with delirium after adjustment for age, gender, BMI, and MMSE score. On the other hand, in the HFpEF group, Barthel index (Odds ratio: 0.390, 95% confidence interval [CI]: 0.20 to 0.78, *p* < 0.01) was significantly associated with delirium after adjustment for age, gender, BMI, EF and MMSE score (Table 3).

### 3.3. Physical Activity and ADL as Markers for Delirium by HF Phenotype

**Figure 1:**
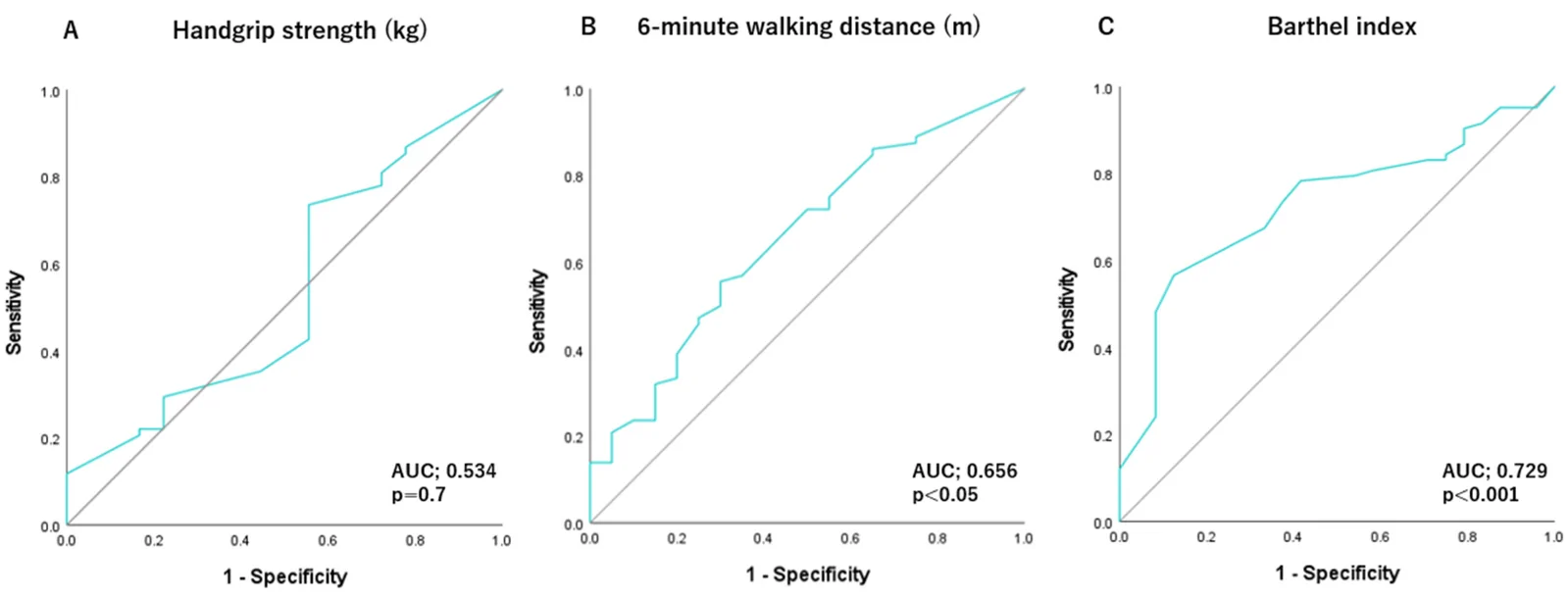
ROC analysis for the discrimination of delirium in HFpEF. (**A**) ROC analysis of handgrip strength (kg) for delirium. (**B**) ROC analysis of 6 min walking distance (m) for delirium. (**C**) ROC analysis of Barthel index for delirium; AUC, area under the curve.

For the HSG, 6MWD and Barthel index, ROC analysis was performed for the identification of delirium in HFpEF (Table 4). Barthel index had the highest value of AUC (Figure 1C), followed by 6MWD (Figure 1B), and HGS (Figure 1A). For discrimination of delirium, AUC value (0.729) and the maximum Youden index (0.441) were obtained at the cut-off value for Barthel index of 67.5. AUC value (0.656) and the maximum Youden index (0.256) were obtained at the cut-off value for 6MWD of 142.5 m.

## 4. Discussion

In this study, older patients with ADHF had a significant association with the relationship between decreased Barthel index and delirium in HFpEF. This reflects the impact of functional decline and ADL decline in HFpEF patients on the development of delirium.

### 4.1. Relationship Between Physical Ability and Delirium in ADHF

Our findings revealed that in older ADHF patients, the group with delirium had a significantly lower value in 6MWD compared to the group without delirium. There are several reasons why delirium is likely to develop in patients with poor exercise capacity. It was reported that neurohumoral factors such as renin–angiotensin system and sympathetic nerve system activations are strongly associated with the decline in exercise capacity [20]. Although the plasma renin activity or sympathetic nervous system activity were not investigated directly, oral MRA administration was found to have a significant negative correlation with delirium in this study, and increased sympathetic nervous activity has been shown in our other HF populations [21,22].

The 6MWD was associated with delirium in the HFpEF group in this study. The cut-off value of 6MWD for delirium determined by the ROC curve was reported to be 345 m in elective cardiac surgery [23]. Although the 6MWD cut-off value was much lower in this study compared to patients who underwent elective cardiac surgery [23], the discrimination via 6MWD for delirium is important because the 6MWD was an easier but valuable measure of exercise capacity even in very old HFpEF patients. These findings suggest that reduced motor ability of the lower limbs is closely related to delirium in very old patients with HFpEF.

### 4.2. Relationship Between ADL and Delirium in ADHF

Our findings revealed that in older ADHF patients, the group with delirium had significantly lower value in Barthel index compared to that without delirium.

Using the Barthel index for ADL enables the assessment of functional outcomes. The coexistence of frailty and the development of delirium in cardiac surgery patients led to an increased mortality one year after surgery [24], and the lower ADL was also a strong predictor of long-term poor outcome in HF patients [25].

In patients with ADHF, dependence is also a risk of delirium [26]. Patients with ADHF are prone to delirium due to a pre-existing factor of cognitive impairment [27]. Cognitive impairment is a well-known risk factor for poor long-term outcomes and is likely associated with HF as a result of shared risk factors such as lower perfusion and rheological abnormalities [28,29]. Although cognitive decline is also involved in delirium, in this study, the association between the Barthel index and delirium remained significant even after adjustment of the MMSE score in HFpEF. Therefore, in this study, the association between lower ADL and delirium appears robust in HFpEF.

### 4.3. HFpEF and Delirium

In the multivariable analysis, only the patients with HFpEF had a significant relationship between lower Barthel index and delirium, while ROC analysis showed that 6MWD and Barthel index were significant markers associated with delirium in HFpEF. Such relationships were not found in HFrEF and HFmrEF.

HFpEF is common in older patients with HF, but pharmacologic therapy for HFpEF is less established than that for HFrEF. Therefore, a multidisciplinary approach other than pharmacological therapy, including interventions for comorbidities and cardiac rehabilitation to maintain physical function, is crucial for the management of HFpEF. In fact, physical frailty was more common in HFpEF compared to HFrEF patients [30]. HFpEF patients had lower HGS and slower gait speed than HFrEF patients [31]. Early cardiac rehabilitation in pre-frail patients is expected to be effective in improving outcomes. In the REHAB-HF study, cardiac rehabilitation effectively improved physical function in older patients with ADHF [32].

The mechanisms participating in HFpEF development are multifactorial, including abnormalities of LV filling and contractility reserve, and morpho-functional derangements associated with skeletal muscles resulting in an abnormal arteriovenous oxygen difference [33]. Skeletal muscle structural and functional impairments represent the peripheral component of exercise intolerance pathophysiology which contributes to the inability to adequately augment skeletal muscle blood flow in response to increasing metabolic demands during exercise [34].

It has been demonstrated that patients with HFpEF have reduced muscle mass, specifically in the lower limbs, and increased intermuscular adipose and altered composition [35]. The biopsy studies revealed decreased mitochondrial-based enzymes and mitochondrial size, a shift in fiber type distribution from slow-twitch, oxidative type I fibers to fast-twitch, glycolytic type IIb fibers [36]. Magnetic resonance spectroscopy demonstrated metabolic abnormalities in leg muscles, and all these skeletal muscle changes were associated with reduced exercise capacity in HFpEF [37]. Barthel index includes items related to lower body physical activity. Therefore, a close relationship between ADL decline and delirium might be observed in this HFpEF population.

A recent report showed that the impairment of lower muscle microcirculation in HFpEF was greater than that in HFrEF patients using near-infrared spectroscopy [38]. These musculovascular associations might be true of the cerebral neurovascular coupling system in patients with HFpEF. The importance of evaluating lower limb strength has been suggested from the perspective of delirium in HFpEF.

In this way, one possible explanation is that patients with HFpEF tend to have a greater burden of geriatric conditions, including frailty and functional decline. Although several established delirium risk factors have been identified, including advanced age, cognitive impairment, comorbidity burden, and illness severity, functional status may provide complementary information regarding an individual’s physiological reserve and overall vulnerability. In this context, the Barthel index may capture dimensions of frailty that are not fully represented by conventional clinical variables. In this point, a lower Barthel index may not directly contribute to the development of delirium. Rather, it may serve as a marker of patient vulnerability. Nevertheless, the present study was not designed to examine these mechanisms, and further prospective research is required to clarify the biological and clinical pathways involved.

### 4.4. Limitations

The present study was observational in nature and did not include detailed assessments of frailty, inflammatory status, or other biological pathways potentially linking functional impairment and delirium. Therefore, mechanistic interpretations should be considered hypothesis-generating. In this study, distinguishing between dyspnea due to HF and delirium at the time of admission was sometimes difficult. Delirium was analyzed as a binary variable (present/absent), and information regarding duration and severity was not available. Because the Barthel index was assessed after stabilization of ADHF, and delirium frequently occurred during the early hospitalization period, the temporal relationship between functional status assessment and delirium onset could not be fully established. Thus, the Barthel index should be interpreted as an associated factor rather than a definitive predictor of delirium. A formal frailty assessment was not performed in this study. Because the Barthel index is closely related to frailty, disability, and cognitive impairment, residual confounding may remain despite adjustment for MMSE. Therefore, the observed association between Barthel index and delirium should be interpreted with caution. The temporal relationship between Barthel index assessment and delirium onset could not be fully established because functional status was evaluated after clinical stabilization rather than at hospital admission. Consequently, delirium may have influenced Barthel index scores in some patients. Therefore, the observed association should be interpreted with caution. Because delirium occurred in 26.5% of participants, the odds ratio between Barthel index and delirium may overestimate the magnitude of association. Therefore, we additionally estimated the prevalence ratio using the ROC-derived cut-off value. The prevalence of delirium was 36.8% among patients with a Barthel index < 67.5 and 9.4% among those with a Barthel index ≥ 67.5. The prevalence ratio (PR) was 3.91 (95% CI 1.59–9.61), corresponding to a prevalence difference of 27.4%. When an adjusted PR (aPR) with 95% confidence intervals was estimated using a generalized linear model with a Poisson distribution, Barthel index (per 1SD) was significantly associated with delirium after adjustment for age, gender, BMI, EF and MMSE score (aPR: 0.608, 95% confidence interval [CI]: 0.35 to 0.95, *p* < 0.05). In both analyses of continuous and binary variables using odds ratios and prevalence ratios, a significant association remained evident for the relationship between Barthel index and delirium in the group with HFpEF. In this study, while 66.15% of patients were hospitalized for initial HF, it cannot be ruled out that the duration elapsed since the first onset of HF might have influenced the Barthel index in the non-initial HF patients. Although the 6MWD values in this study population were low, the baseline value of the 6MWD was 151 m in a previous study similar to our study population [39]. Therefore, the 6MWD values in this study are not considered to widely deviate from previous studies. Cardiac rehabilitation before admission might affect study results. In this study, prehospital preventive cardiac rehabilitation was performed in 17 patients, and no significant differences were found in MMSE scores, HGS, 6MWD, Barthel index, or the presence or absence of delirium between patients who received preventive cardiac rehabilitation and those who did not (all *p* > 0.05). Because this study included consecutive ADHF cases and was not intended to examine patients who received cardiac rehabilitation and those who did not, it is possible that no significant associations were found. Because this is a cross-sectional study, there is no causal relationship between Barthel index and delirium. The number of cases of delirium occurring in the recovery phases was extremely small, making it difficult to examine the types and characteristics of delirium in the recovery phase. Although it is possible that the onset type of HF, treatment intervention and nutrition status might be related to delirium, no significant correlations of new-onset HF, percutaneous coronary intervention, intravenous medication during hospitalization, nutrition status based on geriatric nutritional risk index [40] and controlled nutritional status with delirium [41] were found in this study (all *p* > 0.05). One possible reason for this is the influence of the method of study design, patient enrollment and the small number of cases. Future larger and more methodologically robust studies will warrant drawing conclusions on those issues. The observed predominance of hyperactive delirium differs from the report showing a higher prevalence of hypoactive and mixed subtypes in older hospitalized populations [42]. Although differences in patient characteristics and clinical settings may contribute to this discrepancy, hypoactive delirium is inherently more difficult to recognize in routine practice because patients often present with lethargy, reduced activity, or withdrawal rather than disruptive behaviors. Therefore, under-recognition of hypoactive delirium cannot be excluded, and this possibility should be considered when interpreting the subtype distribution observed in this study. Importantly, risk stratification should not be confused with diagnosis. Although lower Barthel index scores were associated with delirium, the Barthel index is not a diagnostic instrument and cannot replace standardized delirium assessments. Rather, a low score may identify patients who warrant closer monitoring and more comprehensive delirium assessment. When using R software platform (version 4.4.2, R Foundation, Vienna, Austria), in the total population, Barthel index was modeled using restricted cubic splines with four knots at the 5th, 35th, 65th, and 95th percentiles. The overall association between Barthel index and delirium was statistically significant (Wald χ^2^ = 8.71, *p* = 0.033). However, the nonlinear component was not significant (Wald χ^2^ = 2.11, *p* = 0.348), indicating that the relationship was essentially linear in the total population. Therefore, although spline modeling was applied to allow flexible assessment, the association did not show evidence of nonlinearity in the total population. On the other hand, in the HFpEF group, the overall association between Barthel index and delirium was statistically significant (Wald χ^2^ = 14.21, *p* = 0.0026). The nonlinear component was also significant (Wald χ^2^ = 7.77, *p* = 0.0205), indicating that the relationship was not linear in the group with HFpEF. While no evidence of nonlinearity was observed in the overall population, stratified analysis suggested a possible nonlinear association in HFpEF. It has been pointed out that the shape of the spline curve could change depending on the number of knots [43,44]. Therefore, it is unclear whether the relationships shown in this dose–response analysis represent a consistent relationship between Barthel index and delirium in the group with HFpEF.

## 5. Conclusions

Delirium in association with impaired physical activity and ADL is increasingly prevalent in older patients presenting with ADHF. This complex interplay between frailty and delirium further increases the vulnerability of this high-risk population prone to repeat hospitalizations for HF. HFpEF subgroup seems to be at the highest risk and perhaps future interventions should be targeted to cope with both ADL and delirium. Assessment of the Barthel index in older people at high risk for HF and in hospitalized HF patients would help predict the risk of delirium at the next hospitalization.

## Figures and Tables

**Table 1:** Baseline characteristics, physical activity and activity of daily living by delirium.

Characteristics	Delirium (n = 65)	No Delirium (n = 180)	*p* Value
Age (y)	83.3 ± 5.2	82.8 ± 6.3	0.57
Male (%)	49.2	49.4	0.98
New-onset heart failure (%)	66.2	66.1	0.99
Body mass index (kg/m^2^)	21.5 ± 4.7	21.3 ± 4.4	0.76
New York Heart Association class I	-	-	
class II (%)	15.4	17.8	0.66
class III (%)	46.2	37.2	0.21
class IV (%)	38.5	45.0	0.36
Emergency or elective percutaneous coronary intervention (%)	9.23	12.8	0.45
Smoking (%)	23.1	26.1	0.63
Daily alcohol intake (%)	27.7	17.8	0.09
Hypertension (%)	83.1	76.5	0.27
Diabetes mellitus (%)	33.9	33.9	0.99
Medication at baseline			
Beta-blockades (%)	24.6	30.6	0.37
Angiotensin-converting enzyme inhibitors use (%)	9.2	7.8	0.71
Angiotensin II receptor blockers use (%)	43.1	46.1	0.67
Mineralocorticoid receptor antagonists use (%)	6.2	17.2	0.03
Diuretics use (%)	43.1	52.8	0.18
Ischemic etiology (%)	17.5	16.5	0.85
Systolic blood pressure at admission (mmHg)	136 ± 32	142 ± 32	0.26
Diastolic blood pressure at admission (mmHg)	76 ± 17	77 ± 20	0.63
Heart rate at admission (beat per minute)	90 ± 29	88 ± 28	0.58
Brain natriuretic peptide (pg/mL)	543 (242; 905)	521 (224; 912)	0.77
Ejection fraction (%)	47.4 ± 13	48.9 ± 15	0.49
E/e′	20.0 ± 7.8	20.3 ± 7.9	0.82
HFrEF (%)	27.7	33.5	0.40
HFmrEF (%)	32.3	19.6	0.04
HFpEF (%)	40.0	46.9	0.36
Mini–Mental state examination score	19 (14; 25)	25 (21; 27)	<0.001
Handgrip strength (kg)	13.0 (5.0; 18.0)	16.0 (12.0; 20.8)	0.047
6 min walking distance (m)	120 (40.0; 180)	160 (80.0; 229)	0.034
Barthel index	55 (35; 75)	65 (50; 80)	0.013

Data are presented as the mean ± standard deviation, median (interquartile range) or percentages. Analysis of variance for continuous variables or the Kruskal–Wallis test for qualitative variables were used. HFrEF indicates heart failure with reduced ejection fraction; HFmrEF, heart failure with mildly reduced ejection fraction; HFpEF, heart failure with preserved ejection fraction.

**Table 2:** Associations of baseline characteristics, physical activity and activity of daily living with delirium.

Characteristics	Delirium (n = 65)			
	Total (n = 245)	HFrEF (n = 80)	HFmrEF (n = 58)	HFpEF (n = 107)
Age (y)	1.104	1.070	0.966	0.988
Gender (“Male” = 1, “Female” = 0)	0.991	1.273	0.781	1.471
New-onset heart failure (“+” = 1, “−” = 0)	1.002	2.676	0.574	0.848
Body mass index (kg/m^2^)	1.011	1.005	1.018	0.991
New York Heart Association class I	-	-	-	-
class II	Ref	Ref	Ref	Ref
class III	1.433	1.474	0.500	1.225
class IV	0.988	1.081	0.556	0.769
Emergency or elective percutaneous coronary intervention (“+” = 1, “−” = 0)	0.439	1.118	0.001	0.616
Smoking (“+” = 1, “−” = 0)	0.849	1.382	0.816	0.714
Daily alcohol intake (“+” = 1, “−” = 0)	1.771	2.227	1.510	1.695
Hypertension (“+” = 1, “−” = 0)	1.505	1.273	1.286	1.719
Diabetes mellitus (“+” = 1, “−” = 0)	0.998	0.229 *	1.983	1.492
Medication at baseline				
Beta-blockades (“+” = 1, “−” = 0)	0.742	0.538	1.179	0.478
Angiotensin-converting enzyme inhibitors use (“+” = 1, “−” = 0)	1.206	1.133	0.633	3.417
Angiotensin II receptor blockers use (“+” = 1, “−” = 0)	0.884	0.638	1.583	0.769
Mineralocorticoid receptor antagonists use (“+” = 1, “−” = 0)	0.315 *	0.657	0.200	0.001
Diuretics use (“+” = 1, “−” = 0)	0.677	0.538	1.306	0.489
Ischemic etiology (“+” = 1, “−” = 0)	0.694	1.800	0.169	0.694
Systolic blood pressure at admission (mmHg)	0.995	1.001	0.994	0.988
Diastolic blood pressure at admission (mmHg)	0.996	1.004	0.999	0.987
Heart rate at admission (beat per minute)	1.003	1.012	0.992	1.004
Brain natriuretic peptide	1.001	1.001	1.001	1.001
Ejection fraction (%)	0.993	1.001	0.815 *	0.935
E/e′	0.996	0.904 *	1.055	0.967
Mini–Mental state examination score	0.910 ***	0.934	0.844 *	0.871 ***
Handgrip strength (per 1SD)	0.722 *	0.754	0.415 *	0.814
6 min walking distance (per 1SD)	0.706 *	0.873	0.759	0.506 *
Barthel index (per 1SD)	0.713 *	0.921	0.751	0.522 **

Odds ratios by univariate logistic regression analysis. * Significance *p* < 0.05, ** Significance *p* < 0.01, *** Significance *p* < 0.001. HFrEF indicates heart failure with reduced ejection fraction; HFmrEF, heart failure with mildly reduced ejection fraction; HFpEF, heart failure with preserved ejection fraction; SD, standard deviation.

**Table 3:** Multiple logistic regression analysis of physical activity and activity of daily living for delirium stratified by HF phenotype.

Trait	Delirium (n = 65)			
	Total (n = 245)	HFrEF (n = 80)	HFmrEF (n = 58)	HFpEF (n = 107)
Handgrip strength	OR: 0.771	OR: 0.827	OR: 0.756	OR: 0.513
(per 1SD)	(95%CI: 0.52 to 1.14, *p* = 0.19)	(95%CI: 0.44 to 1.55, *p* = 0.56)	(95%CI: 0.24 to 2.40, *p* = 0.76)	(95%CI: 0.18 to 1.46, *p* = 0.51)
6-MWD (per 1SD)	OR: 0.753	OR: 1.060	OR: 1.412	OR: 0.986
	(95%CI: 0.50 to 1.13, *p* = 0.75)	(95%CI: 0.55 to 2.03, *p* = 0.86)	(95%CI: 0.52 to 3.87, *p* = 0.50)	(95%CI: 0.51 to 1.90, *p* = 0.97)
Barthel index	OR: 0.840	OR: 0.852	OR: 2.305	OR: 0.388
(per 1SD)	(95%CI: 0.57 to 1.23, *p* = 0.38)	(95%CI: 0.44 to 1.65, *p* = 0.64)	(95%CI: 0.78 to 6.85, *p* = 0.13)	(95%CI: 0.20 to 0.77, *p* = 0.007)

Each parameter was added separately to the baseline regression models in total population including age, gender, BMI, mineralocorticoid receptor antagonists use and MMSE score, in HFrEF group including age, gender, BMI, diabetes mellitus and E/e′, in HFmrEF group including age, gender, BMI, ejection fraction and MMSE score, in HFpEF group including age, gender, BMI and MMSE score. HFrEF indicates heart failure with reduced ejection fraction; HFmrEF, heart failure with mildly reduced ejection fraction; HFpEF, heart failure with preserved ejection fraction; SD, standard deviation; OR, odds ratio; CI, confidential interval.

**Table 4:** Receiver operator characteristic analysis for the discrimination of delirium in the patients with HFpEF.

Variables	Delirium (n = 65)		
	Area Under the Curve	95% Confidence Interval	*p* Value
Handgrip strength (kg)	0.534	0.379–0.689	0.664
6MWD (m)	0.656	0.522–0.789	0.022
Barthel index	0.729	0.623–0.834	<0.0001

HFrEF indicates heart failure with reduced ejection fraction; 6MWD, 6 min walking distance.

## Data Availability

The original contributions presented in this study are included in the article, further inquiries can be directed to the corresponding authors.

## Abbreviations

The following abbreviations are used in this manuscript:

ADL	Activities of daily living
HF	Heart failure
ADHF	Acute decompensated heart failure
HFpEF	Heart failure with preserved ejection fraction
HGS	Handgrip strength
6MWD	6 min walk distance
MMSE	Mini–Mental state examination
BNP	B-type natriuretic peptide
LV	Left ventricular
EF	Ejection fraction
LVEF	Left ventricular ejection fraction
HFrEF	Heart failure with reduced ejection fraction
HFmrEF	Heart failure with mildly reduced ejection fraction
BMI	Body mass index
ROC	Receiver operating characteristic curves
AUC	Area under the curve
MRA	Mineralocorticoid receptor antagonists
